# Detection and Control of *Pantoea agglomerans* Causing Plum Bacterial Shot-Hole Disease by Loop-Mediated Isothermal Amplification Technique

**DOI:** 10.3389/fmicb.2022.896567

**Published:** 2022-05-25

**Authors:** Ran Shu, Xianhui Yin, Youhua Long, Jun Yuan, Houyin Zhou

**Affiliations:** ^1^Engineering and Technology Research Center of Kiwifruit, Guizhou University, Guiyang, China; ^2^Institute of Crop Protection, Guizhou University, Guiyang, China

**Keywords:** *Pantoea agglomerans*, plum bacteria shot-hole disease, loop-mediated isothermal amplification, control, detection

## Abstract

Plum bacterial shot-hole caused by *Pantoea agglomerans* (*P*. *agglomerans*) is one of the primary bacterial diseases in plum tree planting areas, resulting in abnormal growth of plum trees and severe economic losses. Early diagnosis of *P*. *agglomerans* is crucial to effectively control plant diseases. In this study, loop-mediated isothermal amplification (LAMP) analysis for genome-specific gene sequences was developed for the specific detection of *P*. *agglomerans*. We designed the LAMP primers based on the *gyrB* gene of *P. agglomerans*. The best reaction system was 0.2 μmol·L^−1^ for outer primer F3/B3 and 1.6 μmol·L^−1^ for inner primer FIP/BIP. The LAMP reaction was optimal at 65°C for 60 min based on the color change and gel electrophoresis. This technology distinguished *P. agglomerans* from other control bacteria. The detection limit of the LAMP technology was 5 fg·μl^−1^ genomic DNA of *P*. *agglomerans*, which is 1,000 times that of the traditional PCR detection method. The LAMP technology could effectively detect the DNA of *P. agglomerans* from the infected leaves without symptoms after indoor inoculation. Furthermore, the LAMP technology was applied successfully to detect field samples, and the field control effect of 0.3% tetramycin after LAMP detection reached 82.51%, which was 7.90% higher than that of conventional control. The proposed LAMP detection technology in this study offers the advantages of ease of operation, visibility of results, rapidity, accuracy, and high sensitivity, making it suitable for the early diagnosis of plum bacteria shot-hole disease.

## Introduction

Plums are widely distributed fruit trees globally (Maatallah et al., 2015), wild or cultivated in China, the United States, Japan, and the European countries (Wu et al., 2018; Wei et al., 2021). Studies have shown that various plum diseases, including brown rot, bacterial shot-hole, and a red spot among others (Kleina et al., 2018), often cause severe damage to the infected trees and often kill them even. *P. agglomerans*, a gram-negative bacterium (Moloto et al., 2020) is a dominant pathogen causing bacterium shot-hole disease in major plum-growing areas of Guizhou Province, China. In recent years, the plum bacterial shot-hole disease has occurred in Xiuwen County, Huishui County, Yinjiang County, and other areas of Guizhou Province. Usually, this pathogenic bacterium infects branches, leaves, or fruits, resulting in abnormal absorption and transportation of water and mineral nutrients in plums, and eventually causing plant death (Li et al., 2020; Tong et al., 2020). *Pantoea agglomerans* could also cause other plant diseases, including but not limited to bacterial soft rot of cabbage (Guo et al., 2019), blight disease on Pepino melon (She et al., 2021), necrotic disease in *Ziziphus jujuba* (She et al., 2019), and brown apical necrosis of Walnut (Yang et al., 2011). Furthermore, *P. agglomerans* can cause some occupational diseases in humans (Büyükcam et al., 2018) and infect some animals (Zhang et al., 2016). In general, the effect of using control measures is unsatisfactory after the occurrence of symptoms of bacterial infection. Thus, rapid and specific detection of *P. agglomerans* in advance is visible symptoms crucial to prevent its spread.

The conventional PCR, multiplex PCR, and real-time quantitative PCR have been widely applied to the specific diagnosis of various *Pantoea* species. For instance, Baek et al. (2018) used conventional PCR to diagnose *Pantoea stewartii* subsp. *stewartii* accurately in plants with a detection limit of 2 pg/μl genomic DNA. Kini et al. (2021a,b) designed multiplex PCR primers based on the whole genome sequence of *Pantoea* and detected *P. agglomerans, P. ananatis*, and *P. stewartii* growing in rice seeds with a detection threshold at 0.5 ng/μl genomic DNA. Tambong et al. (2010) established a TaqMan-based real-time PCR assay for the detection and identification of *P. stewartii* in maize, which targets the *cpsD* gene and could specifically detect *P*. *stewartii* in maize leaves and seeds with a minimum of 1 pg/μl of genomic DNA. However, with the rapid development of molecular detection technology, it has become possible to establish a more rapid, accurate, highly sensitive, and easy-to-operate detection technology of pathogens. The loop-mediated isothermal amplification, as an isothermal nucleic acid amplification technology suitable for genetic diagnosis, was published by Notomi (2000) in 2000. This technology can specifically amplify target DNA fragments by using highly active strand displacement DNA polymerase (Bst DNA polymerase) at constant temperature (60°C~65°C) (Notomi et al., 2015), which has been widely applied to diagnose pathogens owing to its advantages of rapidity, low cost, high sensitivity, and strong specificity (Ortega et al., 2018). Katoh et al. (2020) diagnosed *Fusarium oxysporum* causing strawberry wilt by using LAMP technology with a detection threshold at 100 pg·μl^−1^, which could also distinguish *Fusarium oxysporum* from several other fungal pathogens on strawberries. Moreover, a previous study has shown that the pathogen *Xanthomonas arboricola* pv. *pruni* could be detected within 15 min by using real-time LAMP (Li et al., 2019). Tegli et al. (2020) established a LAMP method for detecting *Curtobacterium flaccumfaciens* pv. *flaccumfaciens* after 30 min and the detection limit was reduced to 4 fg·μl^−1^ of genomic DNA. In short, all these LAMP methods exhibited the advantages of strong specificity, high sensitivity, and time saving. The LAMP reaction products can be detected not only by gel electrophoresis but also observed by naked eyes after being stained with SYBR Green I (Yan et al., 2019), hydroxy naphthol blue (HNB) (Goto et al., 2010; Ren et al., 2021) or calcein (Tomita et al., 2008). Thus far, no studies have been conducted on the rapid detection of *P. agglomerans* by using the LAMP method. In this study, we established, for the first time, a LAMP method for rapidly detecting the *P. agglomerans* in plum leaves and stems. Furthermore, we also evaluated the control effect of several antibiotics against plum bacterial shot-hole by combining the LAMP technology with field control measures.

## Materials and Methods

### Bacterial Strains and Culture Conditions

Plum leaves with typical bacterial shot-hole and branches were sampled from the orchard in Guizhou Province, China. By using the tissue isolation method (She et al., 2021), 18 *P. agglomerans* strains were obtained from infected leaves and branches. The pathogenicity test (She et al., 2021) showed that the HXFJ strain was the most pathogenic. Therefore, strain HXFJ was selected for the LAMP assay. The morphological and phylogenetical analyses of strain HXFJ were shown in Supplementary Figures S1, S2. All strains used in this study were cultured on a nutrient agar (NA) medium at 28°C. The remaining test bacterial strains used in this study are shown in Table 1, No. 1–3 strains were presented by the Guizhou Academy of Agricultural Sciences, and No. 4–10 strains were isolated and preserved by our laboratory.

**Table 1:** Test strains and sources.

	**Bacterial strains**	**Host**	**Strain source**
1	*Pectobacterium carotovorum*	Konjac	Guizhou Academy of Agricultural Sciences
2	*Rastonia solanacearum*	Tomato	Guizhou Academy of Agricultural Sciences
3	*Xanthomonas axonopodis* pv. *citri*	Tangerine	Guizhou Academy of Agricultural Sciences
4	*Pseudomonas syringae* pv. *actinidiae*	Kiwifruit	Xiuwen County, Guizhou Province
5	*Pseudomonas viridiflava*	Kiwifruit	Xiuwen County, Guizhou Province
6	*Pantoea stewartii*	Corn	Kaiyang County, Guizhou Province
7	*Pantoea ananatis*	Plum	Xifeng County, Guizhou Province
8	*Xanthomonas campestris* pv. *mangiferaeindicae*	Mango	Luodian Country, Guizhou Province
9	*Xanthomonas euvesicatoria*	Chili	Zunyi City, Guizhou Province
10	*Xanthomonas arboricola* pv. *juglandis*	Walnut	Xiuwen County, Guizhou Province

### DNA Extraction

The selected isolates were transferred into the nutrient broth (NB: glucose 10.0 g/L, protein 10.0 g/L, beef cream 3.0 g/L, yeast cream 1.0 g/L, and pH 7.0 ~7.2) medium and placed on a shaker (120 rpm at 28°C) until the bacterial solution became turbid. The bacterial solution was transferred to Eppendorf tubes for DNA extraction. The extraction of DNA was performed according to the bacterial genomic DNA extraction kit (Beijing Solarbio Science & Technology Co., Ltd., Beijing, China) as per the manufacturer's instructions.

### Primer Design

The *gyrB* gene sequence was selected for designing LAMP primers by using the Primers Explorer V5 software program (available online at http://primerexplorer.jp/e/). The LAMP primer set (Nam et al., 2020) (*gyrB*-2) has six primers (Supplementary Figure S3), including outer primers F3 and B3, inner primers FIP and BIP (FIP containing F1 and F2, BIP containing B1 and B2), loop primers LF and LB (Supplementary Table S1).

### PCR Assay

The PCR amplification was performed in a Thermal Cycler (iCycler, BIO-RAD, USA) in a total volume of 25 μl containing 2 × Taq PCR Master Mix [(Sangon Biotech Co. Ltd. (Shanghai, China)] 12.5 μl, 10 μM primer F3 1 μl, 10 μM primer B3 1 μl, DNA 1 μl, and ddH_2_O 9.5 μl. Reaction mixes were amplified at 94°C for 5 min, 34 cycles of 30 s at 94°C, 60 s at 55°C, and 30 s at 72°C. A final extension was accomplished for 10 min at 72°C. After amplification, the mixture was examined by 1.0% agarose gel electrophoresis.

### LAMP Assay

The LAMP reaction adopts a 25 μl system: 10 × Isothermal Amplification Buffer 2.5 μl, 6 mM MgSO_4_ [New England Biolabs (Beijing) LTD., Beijing, China], 1.4 mM dNTP Mix (10 mM) Sangon Biotech Co. Ltd. (Shanghai, China), 0.2 μM each of the F3 and B3, 1.6 μM each of primer FIP and BIP, 0.4 μM each of primer LB and LF, Bst 2.0 DNA Polymerase (8,000 U/ml) [New England Biolabs (Beijing) Ltd., Beijing, China] 1 μl, DNA sample 1 μl, ddH_2_O makes up 25 μl. The mixture was incubated at 65°C for 60 min. The reaction was terminated by heating at 80°C for 5 min and the products were separated by electrophoresis in 1.0% agarose gel. After the reaction product cooling, add 1 μl of 1/10 diluted original SYBR Green I (Beijing Solarbio Science & Technology Co., Ltd., Beijing, China) solution for color reaction.

### Optimization of LAMP System

The LAMP reaction system (the final concentration of FIP/BIP and F3/B3) and reaction conditions (temperature and time) were optimized, respectively. All treatments were performed in triplicates. The final concentration of FIP/BIP was set to 0.8, 1.2, 1.6, 2.0, and 2.4 μM. The final concentration of F3/B3 was set to 0, 0.1, 0.2, 0.3, and 0.4 μM. The reaction temperature was set to 59, 61, 63, 65, 67, and 69°C. The reaction time was set to 20, 30, 40, 50, 60, 70, and 80 min. The concentration of other components and reaction conditions was performed as per the LAMP reaction system mentioned in section LAMP Assay.

### Sensitivity and Specificity Assay

All alluded procedures were repeated three times. The genomic DNA of *P*. *agglomerans* was serially diluted 10 times using ddH_2_O with the concentrations ranging from 50 ng·μl^−1^ to 0.5 fg·μl^−1^. After that, the serially diluted genomic DNA was used to determine the sensitivity of the LAMP primers. To evaluate the specificity of the selected primers, *P*. *agglomerans* and 10 pathogens (Table 1) were selected for further test. The reaction results were visualized by adding 1 μl of 10 times diluted SYBR Green to the reaction tube. A positive reaction was green, whereas the color remained orange to the naked eye for a negative response.

### Practicality Assay

To evaluate the versatility and practicability of LAMP primers, nine samples from Huishui, Kaiyang, and Xiuwen regions in Guizhou Province were subjected to LAMP reaction with ddH_2_O as a negative control. To test the effect of LAMP on the detection of infected tissue, the overnight cultured *P*. *agglomerans* suspension was inoculated into fresh plum leaves. After being moisturized for 3, 6, 9, 12, and 24 h, the leaves were rinsed with sterilized ultrapure water and used uninfected leaves as negative sample control. The tissue of leaves (samples were surface sterilized.) was cut and the DNA was extracted using Plant Genomic DNA Extraction Kit (Beijing Solarbio Science & Technology Co., Ltd., Beijing, China). Subsequently, the leaves' genomic DNA were used as a DNA template for LAMP detection. *P*. *agglomerans* DNA was used as a positive control. Negative control was performed using ddH_2_O.

### Application of LAMP Technology in the Field Test of Control Plum Bacterial Shot-Hole

The field experimental site is located in the plum orchard in Xiuwen County, Guiyang City, Guizhou Province, China.

On 30 April 2021, the plum leaves and branches were collected from the orchard. The genomic DNA was extracted using Plant Genomic DNA Kit [Tiangen BioTech (Beijing) Co., Ltd.] as per the manufacturer's instructions. Then, the LAMP method established in our study was carried out to detect whether the sample carries *P. agglomerans* or not. After confirming that the samples were infected with *P. agglomerans* (at the same time, taking microbial analysis as a reference method, it was determined to be *Pantoea agglomerans*), further field control trials against plum bacterial shot-hole were performed on 31 April 2021. In brief, 0.3% tetramycin aqueous solution (AS) 600 times dilution liquid, 2% kasugamycin·tetramycin soluble concentrate (SL) 600 times dilution liquid, and 2% zhongshengmycin·tetramycin soluble concentrate (SL) 600 times dilution liquid were selected for field control against plum bacterial shot-hole by foliar spray. The negative control was performed by spraying the same amount of water.

#### Conventional Control

The same experiment was performed again when the plum leaves exhibited an obvious bacterial shot-hole on 7 May 2021. All experiments were performed in triplicate and investigated after 14 days.

The disease severity grading standards (leaves) are as follows (Bálint et al., 2016): Grade 0, no disease spots; Grade 1, the lesion area accounts for <5% of the total leaf area; Grade 2, the diseased spot area accounts for 6~15% of the total leaf area; Grade 3, the lesion area accounts for 16~25% of the total leaf area; Grade 4, the lesion area accounts for 26~50% of the total leaf area; Grade 5, the lesion area accounts for more than 50% of the total leaf area. The disease index and control effect of plum bacterial shot-hole were calculated according to the following formula. Disease index = (number of diseased leaves × disease grade index)/(the total number of investigated leaves × 5) × 100; Control effect = (disease index of the control group–disease index of treatment group)/disease index of control group × 100.

## Results

### Result of Optimization of LAMP System

**Figure 1:**
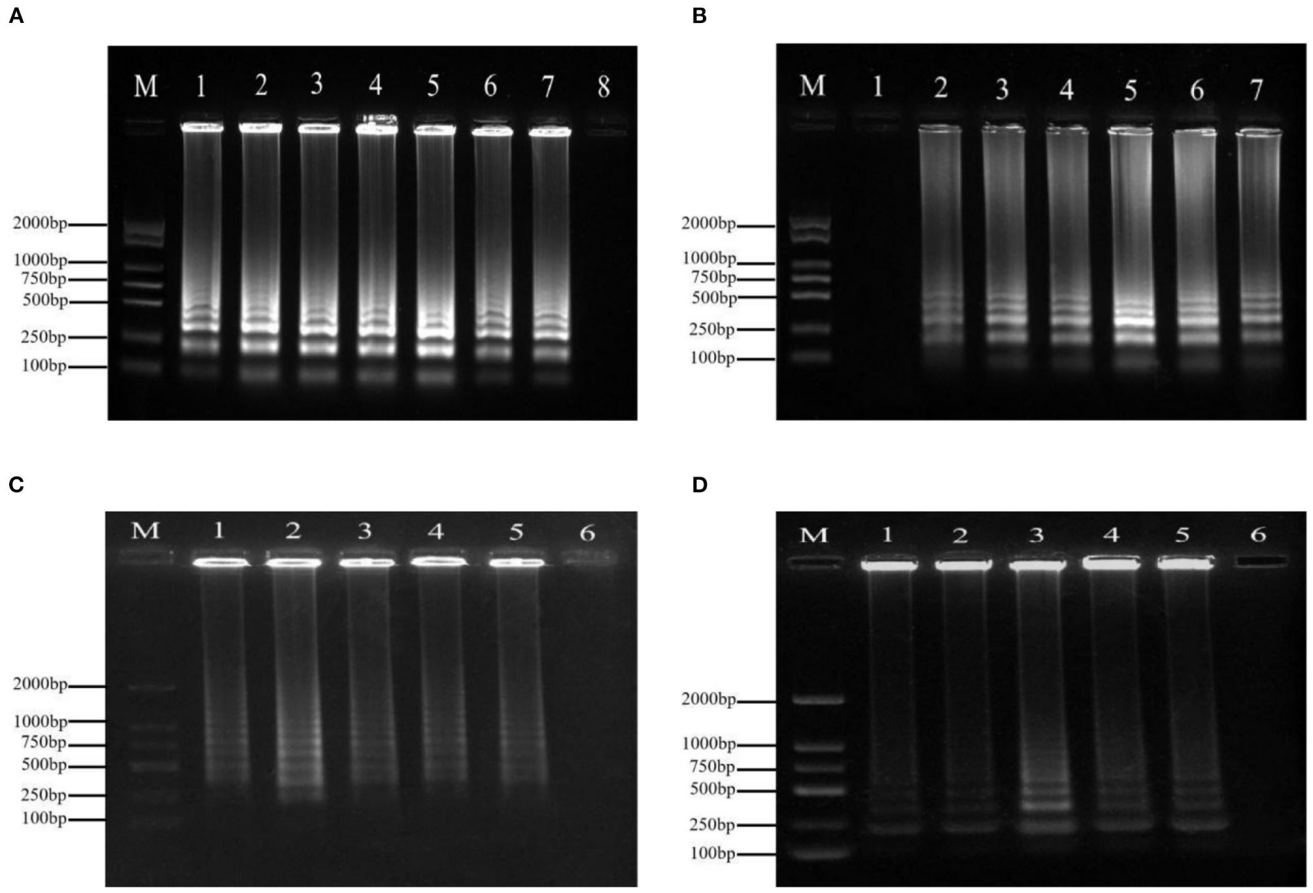
The optimization of the LAMP for the detection of *P*. *agglomerans*. **(A)** Time. 1: 20 min; 2: 30 min; 3: 40 min; 4: 50 min; 5: 60 min; 6: 70 min; 7: 80 min; and 8: negative control (ddH_2_O); **(B)** Temperature. 1: Negative control (ddH_2_O); 2: 59°C; 3: 61°C; 4: 63°C; 5: 65°C; 6: 67°C; and 7: 69°C; **(C)** Outer primers concentration (inner primer concentration: 1.6 μmol·L^−1^). 1: 0.1 μmol·L^−1^ 2: 0.2 μmol·L^−1^; 3: 0.3 μmol·L^−1^; 4: 0.4 μmol·L^−1^; 5: 0.5 μmol·L^−1^; and 6: Negative control (ddH_2_O); **(D)** Inner primer concentration (outer primers concentration: 0.2 μmol·L^−1^). 1: 0.8 μmol·L^−1^ 2: 1.2 μmol·L^−1^; 3: 1.6 μmol·L^−1^; 4: 2.0 μmol·L^−1^; 5: 2.4 μmol·L^−1^; and 6: negative control (ddH_2_O).

Variations in temperature, time, and the primer concentrations of F3/B3 and FIP/BIP were studied to evaluate the effect on the amplifying reaction. The typical ladder-like banding pattern of amplification products was visible after agarose gel electrophoresis at incubation temperatures ranging from 59to 69°C (Figure 1B). The optimum reaction time was 60 min (Figure 1A). Ladder-like bands could be obtained by LAMP amplification when the final concentrations of FIP/BIP and F3/B3 were 0.8–2.4 and 0.1–0.5 μmol·L^−1^, respectively. When the concentration of FIP/BIP and F3/B3 increased to 1.6 and 0.2 μmol·L^−1^, respectively, the amplified bands were the clearest and brightest (Figures 1C,D).

### Evaluation of Sensitivity and Specificity

**Figure 2:**
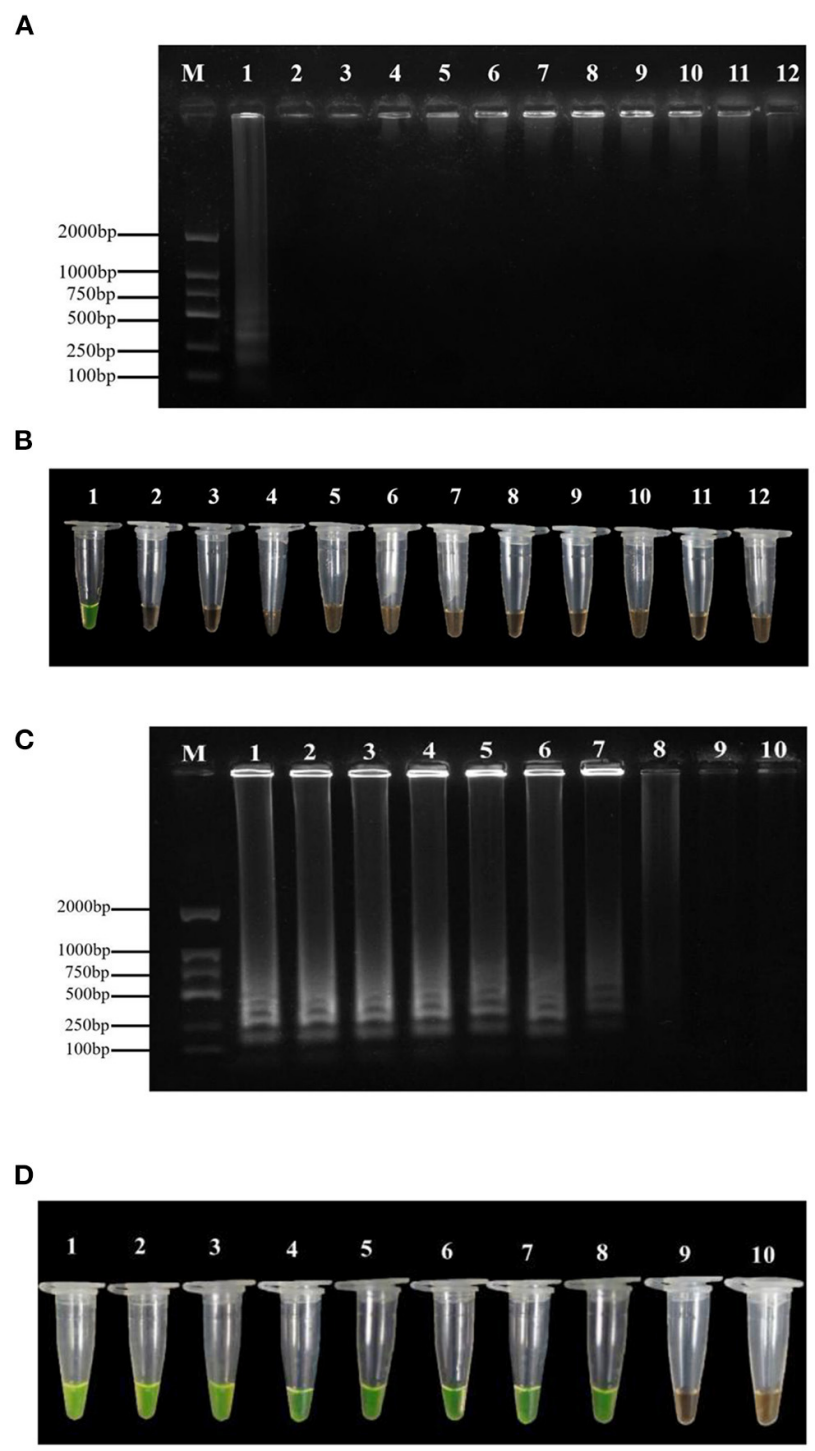
Specificity and sensitivity tests of the LAMP assay. **(A)** Agarose gel electrophoresis analysis of the LAMP products. M: DL1500 DNA marker; 1: positive control; 2–11: the DNA template of *P. carotovorum, R. solanacearum, P. viridiflava, Pseudomonas syringae pv. Actinidiae, P. stewartii, P. ananatis, Xanthomonas axonopodis pv. citri, Xanthomonas campestris pv. Mangiferaeindicae, X. euvesicatoria and Xanthomonas arboricola pv. Jugland* is selected were respectively; 12: negative control (ddH_2_O). **(B)** Assessment based on SYBR Green I visualization of color change. **(C)** Agarose gel electrophoresis analysis of the LAMP products. M indicates a DL1500 DNA marker; numbers 1 to 9 indicate the DNA concentration of 50, 5, 0.5 ng·μl^−1^, 50, 5, 0.5 pg·μl^−1^, 50, 5, and 0.5 fg·μl^−1^, respectively; number 10 indicates negative control (ddH_2_O). **(D)** Assessment based on SYBR Green I visualization of color change.

The results of the specificity assay showed that the LAMP test of *P*. *agglomerans* exhibited yellow-green. In contrast, the LAMP test of the other ten pathogenic bacteria and negative control showed orange (Figure 2B). The amplified products detected by 1% agarose gel electrophoresis showed that the positive control presented the typical ladder-like banding pattern, while the negative control and the other 10 bacteria did not (Figure 2A). Therefore, the LAMP primers were highly specific to *P*. *agglomerans* and the minimum LAMP detection limit was 5 fg·μl^−1^ (Figures 2C,D), which was 1,000 times higher than that of conventional PCR, with the minimum detection concentration of 5 pg·μl^−1^ (Supplementary Figure S4).

### Result of Practicality Assay

**Figure 3:**
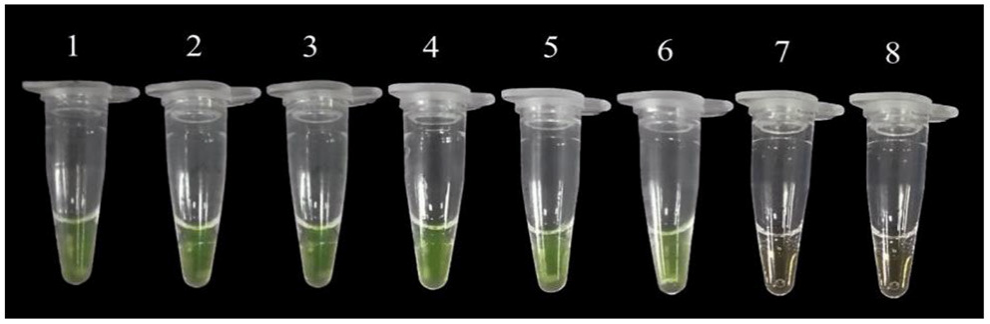
The detection of P. agglomerans in the diseased plant by LAMP. 1: Positive control; 2~6: Inoculation time of 3, 6, 9, 12, 24 h; 7: Healthy plum leaf tissue; 8: Negative control (ddH_2_O).

**Figure 4:**
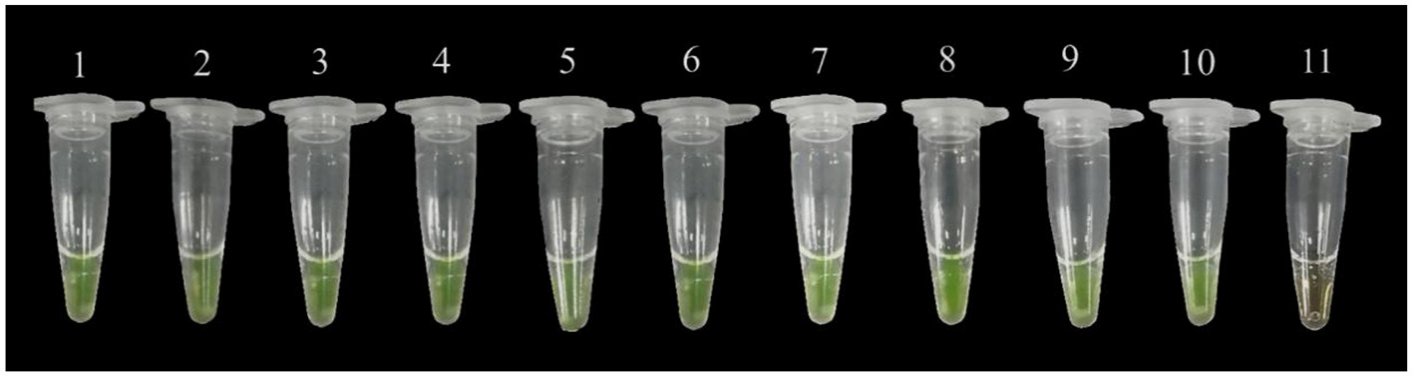
Field sample testing. 1~3: gDNA isolated from plum leave from Huishui; 4~6: gDNA isolated from plum leave from Xiuwen; 7~9: gDNA isolated from plum leave from Kaiyang; 10: Positive control; 11: Negative control (ddH_2_O).

From the positive reaction results we observed in 200 μl tubes, the LAMP method could detect the bacterial DNA in leaves 3, 6, 9, 12, and 24 h after inoculation with *P. agglomerans* (Figure 3), which further confirmed the accuracy of this LAMP technology. In addition, the LAMP primers were selected to amplify the DNA of nine suspected disease samples collected from Huishui, Kaiyang, and Xiuwen, Guizhou Province. All the LAMP amplification results exhibited yellow–green positive reactions (Figure 4), indicating that the LAMP primers had excellent practicability.

### Application of LAMP Technology for Controlling Plum Bacterial Shot-Hole Disease

Compared with the conventional disease control, the combination of LAMP detection technology and field control could improve the control effect against plum bacterial shot-hole disease (Tables 2, 3). Among the three antibiotics, 0.3% tetramycin showed an excellent control effect of 82.51%, which was 7.90% higher than that of conventional control, followed by 2% kasugamycin·tetramycin with a control effect increased by 9.51%. These results suggested that the combination of LAMP technology and field control is an effective method for better management of plant diseases.

**Table 2:** The control effect after detection using the LAMP method in advance.

**Treatment**	**Disease index**	**Control efficiency**
0.3% Tetramycin AS	1.39	82.51 ± 0.61b
2% kasugamycin·tetramycin SL	1.83	77.05 ± 1.27c
2% zhongshengmycin·tetramycin SL	2.22	71.98 ± 1.37d
Control	7.95	-

*Different lowercase letters represented a significant difference (P < 0.05)*.

**Table 3:** The control effect of the conventional control method.

**Treatment**	**Disease** **index**	**Control efficiency**
0.3% Tetramycin AS	2.62	76.47 ± 1.58a
2% kasugamycin·tetramycin SL	3.32	70.36 ± 1.45b
2%zhongshengmycin·tetramycin SL	3.99	64.24 ± 1.254c
Control	11.18	

*Different lowercase letters represented a significant difference (P < 0.05)*.

## Discussion

In this study, six specific primers were designed using the *gyrB* gene of *P*. *agglomerans* as the target. Prameela et al. (2017) designed the LAMP primers from the *gyrB* gene, which could specifically detect race 4 *R. pseudosolanacearum* infecting *Zingiberaceae* plants. At the same time, combined with SYBR Green I color rendering, a visual LAMP detection method was established. The detection method could effectively detect the target DNA fragment within 20 to 80 min. The detection time decreased and detection speed improved by using this method.

Sensitivity is one of the most crucial factors in molecular detection technology. The higher the sensitivity, the more conducive to detecting target pathogens from micro samples (Duan et al., 2014a,b; Notomi et al., 2015). The high sensitivity of LAMP technology provides more accurate guidance and reference for disease detection and prevention (Lee et al., 2017). Compared with the conventional PCR, LAMP technology has more advantages in sensitivity (Le and Vu, 2017). For example, the detection sensitivity of LAMP technology on *Sclerotinia sclerotiorum* and *Phytophthora melonis* is 1,000 times higher than that of PCR (Chen et al., 2013; Duan et al., 2014b). The LAMP method established in this test showed high sensitivity. The minimum detection limit of DNA was 5 fg·μl^−1^, which was 10 times more sensitive than the LAMP detection method established by Kini et al. (2021a,b) and 1,000 times higher than that of conventional PCR.

In addition, the LAMP technology has high specificity, which is easier to distinguish target pathogens from non-target pathogens in the detection process, making the detection results more accurate and reliable. A total of ten plant pathogens were used as non-target bacteria in the specificity assay of LAMP. The LAMP detection method could effectively distinguish *P*. *agglomerans* from other pathogens, including *P. carotovorum, P. viridiflava, Pseudomonas syringae* pv. *Actinidiae*, and *P. ananatis*, among others.

Identifying plant diseases, especially bacterial diseases, is the premise of effective and accurate prevention of plant diseases in a complex environment (Ali et al., 2019). In general, it is hard to observe typical symptoms in the early stage of bacterial infection, which causes people to miss the optimal period of controlling plant diseases. Based on this, the LAMP technology is a good strategy for the early diagnosis of bacterial diseases, which can prevent plant disease and pathogens during the preliminary stage and aids in health control and yield optimization without depending on the pest and pesticides usage (Le and Vu, 2017). Xiao and Li (2021) detected the soft rot pathogen of *Dendrobium officinale* by the LAMP method and then tested the control effect of several fungicides. After 14 days of spraying, the synergistic efficacy of the two fungicides (pyraclostrobin and picoxystrobin) reached 82.39%. In this study, compared with conventional disease control, the combination of LAMP detection technology and chemical control could improve the effect of preventing disease. Among the three antibiotics, 0.3% tetramycin showed an excellent control effect of 82.51%, which was 7.90% higher than that of conventional control. These results suggested that the combination of LAMP technology and chemical control is an effective method for better management of plant diseases.

## Conclusions

In this study, three pairs of primers were designed according to the sequence of *P. agglomerans gyrB*, and SYBR Green I was used as the indicator to establish the LAMP visual rapid detection method for the pathogen of plum bacterial shot-hole disease (*P. agglomerans*). This method has strong specificity, high sensitivity, short inspection time, and strong practicability, which can provide a theoretical basis for the early and rapid diagnosis of *Pantoea agglomerans* in the plum production.

## Data Availability Statement

The original contributions presented in the study are included in the article/Supplementary Material, further inquiries can be directed to the corresponding author.

## Author Contributions

RS: formal analysis, writing original draft, and data curation. XY: funding acquisition, conceptualization and writing review, and editing. YL: methodology and validation. JY: visualization. HZ: investigation. All the authors have read and agreed to the published version of the manuscript.

## Funding

This research was financially supported by the Science and Technology Project of Guizhou Province (grant no. QKHZC2406-2019) and the 13th Batch of Guizhou Outstanding Young Scientific and Technological Talents Project (grant no. QKHPTRC5614-2021).

## Conflict of Interest

The authors declare that the research was conducted in the absence of any commercial or financial relationships that could be construed as a potential conflict of interest.

## Publisher's Note

All claims expressed in this article are solely those of the authors and do not necessarily represent those of their affiliated organizations, or those of the publisher, the editors and the reviewers. Any product that may be evaluated in this article, or claim that may be made by its manufacturer, is not guaranteed or endorsed by the publisher.
