# Supplementary material for: Detection and Control of Pantoea agglomerans Causing Plum Bacterial Shot-Hole Disease by Loop-Mediated Isothermal Amplification Technique
Source: Front Microbiol. 2022 May 25;13:896567. doi: 10.3389/fmicb.2022.896567 (PMC9175033; doi:10.3389/fmicb.2022.896567)
Supplement: Supplementary file 1 [file Table_1.DOCX]

S1 Table LAMP primer sequences

| Primer name | Primer sequences（5`~3`） |
| --- | --- |
| F3 | GGCGTTTGTTGAGTACCTCA |
| B3 | TGGCCTTCTTGCTGTAACC |
| FIP | GTTCCACTGCAGCGCCACTTCGATTCACCCTACCGTGTTC |
| BIP | AACAACATCCCACAGCGCGACATTCAGGGTACGGGTCATC |
| LF | ACACCAATGCCATCTTTCTCGGT |
| LB | TACACACCTCGCCGGTTTCC |
