# Supplementary figures and images for: Detection and Control of Pantoea agglomerans Causing Plum Bacterial Shot-Hole Disease by Loop-Mediated Isothermal Amplification Technique
Source: Front Microbiol. 2022 May 25;13:896567. doi: 10.3389/fmicb.2022.896567 (PMC9175033; doi:10.3389/fmicb.2022.896567)

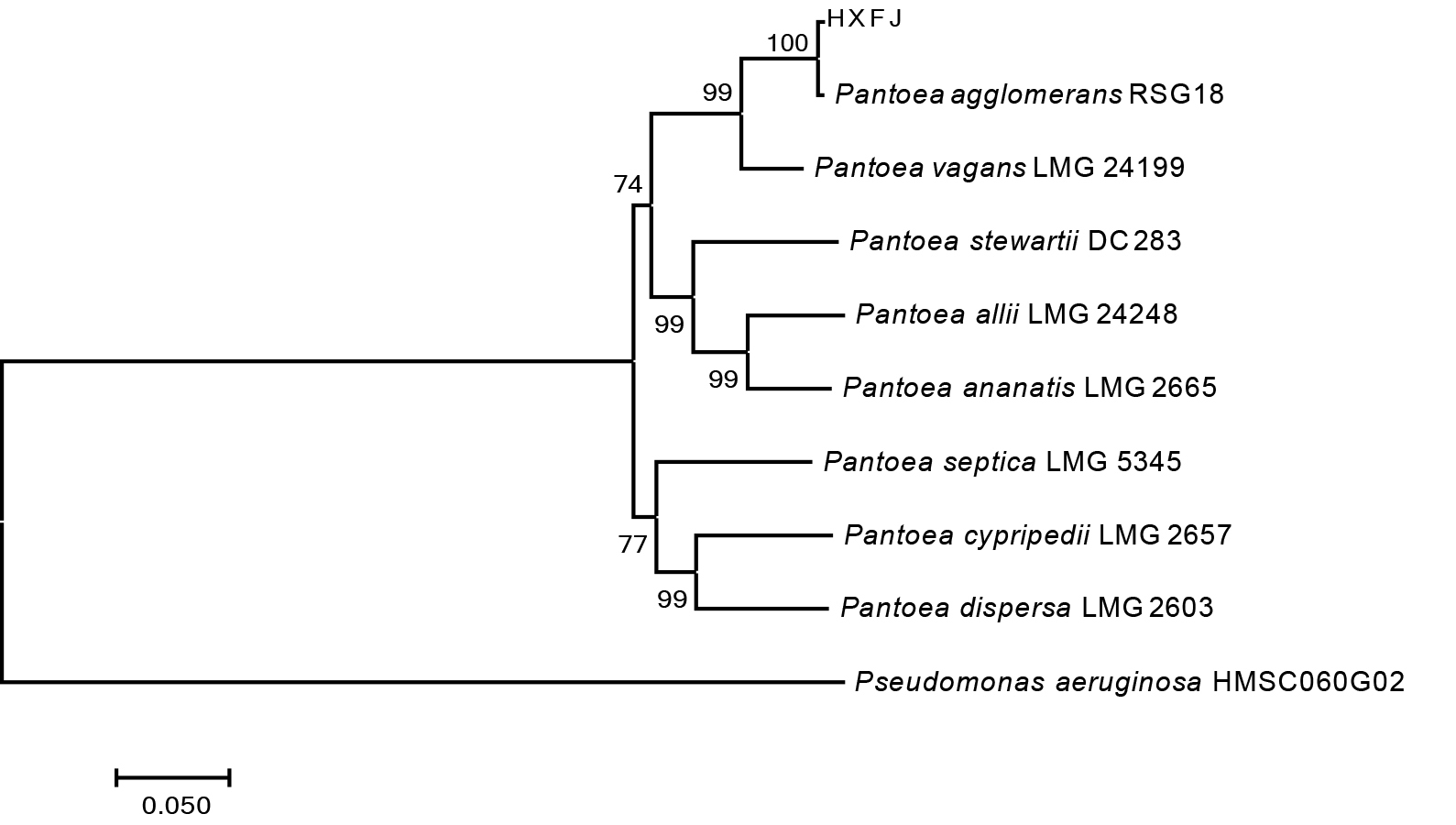

Supplement: Supplementary file 2 [file Image_1.JPEG]

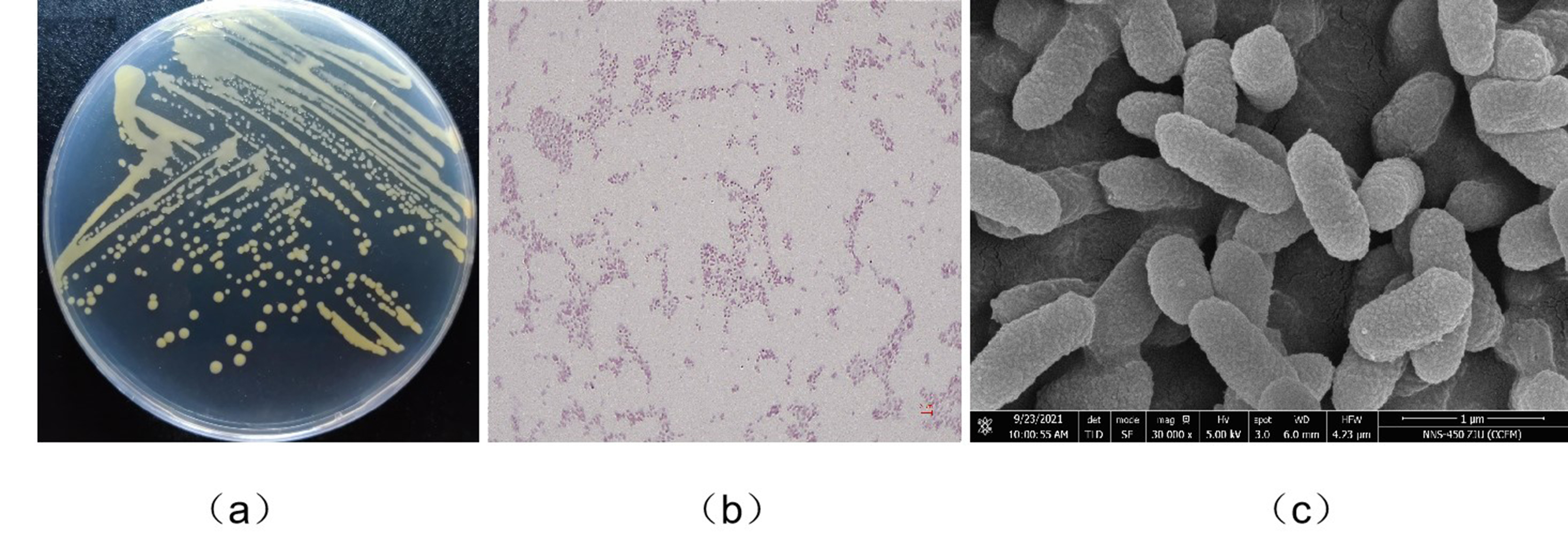

Supplement: Supplementary file 3 [file Image_2.JPEG]

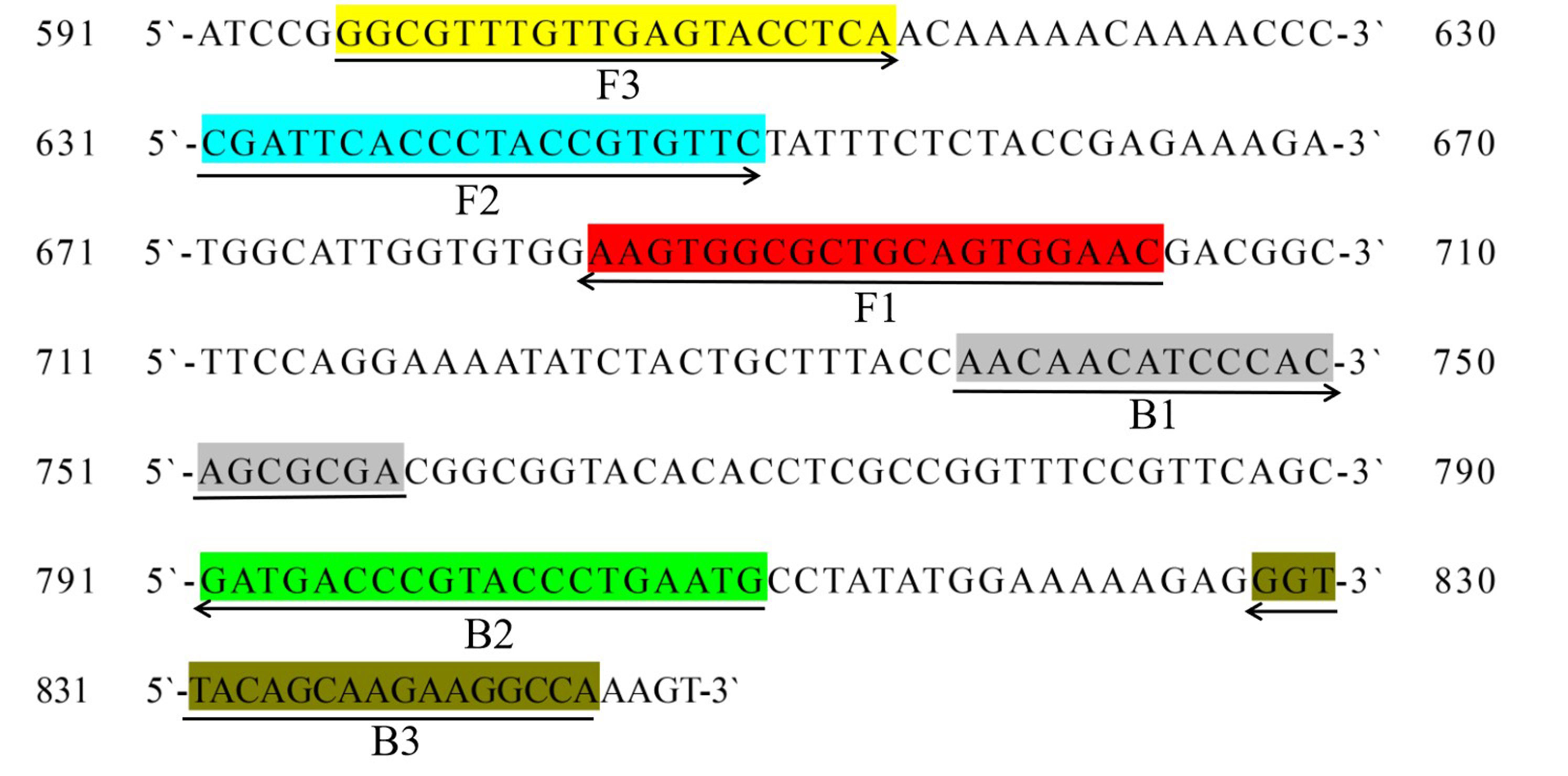

Supplement: Supplementary file 4 [file Image_3.JPEG]

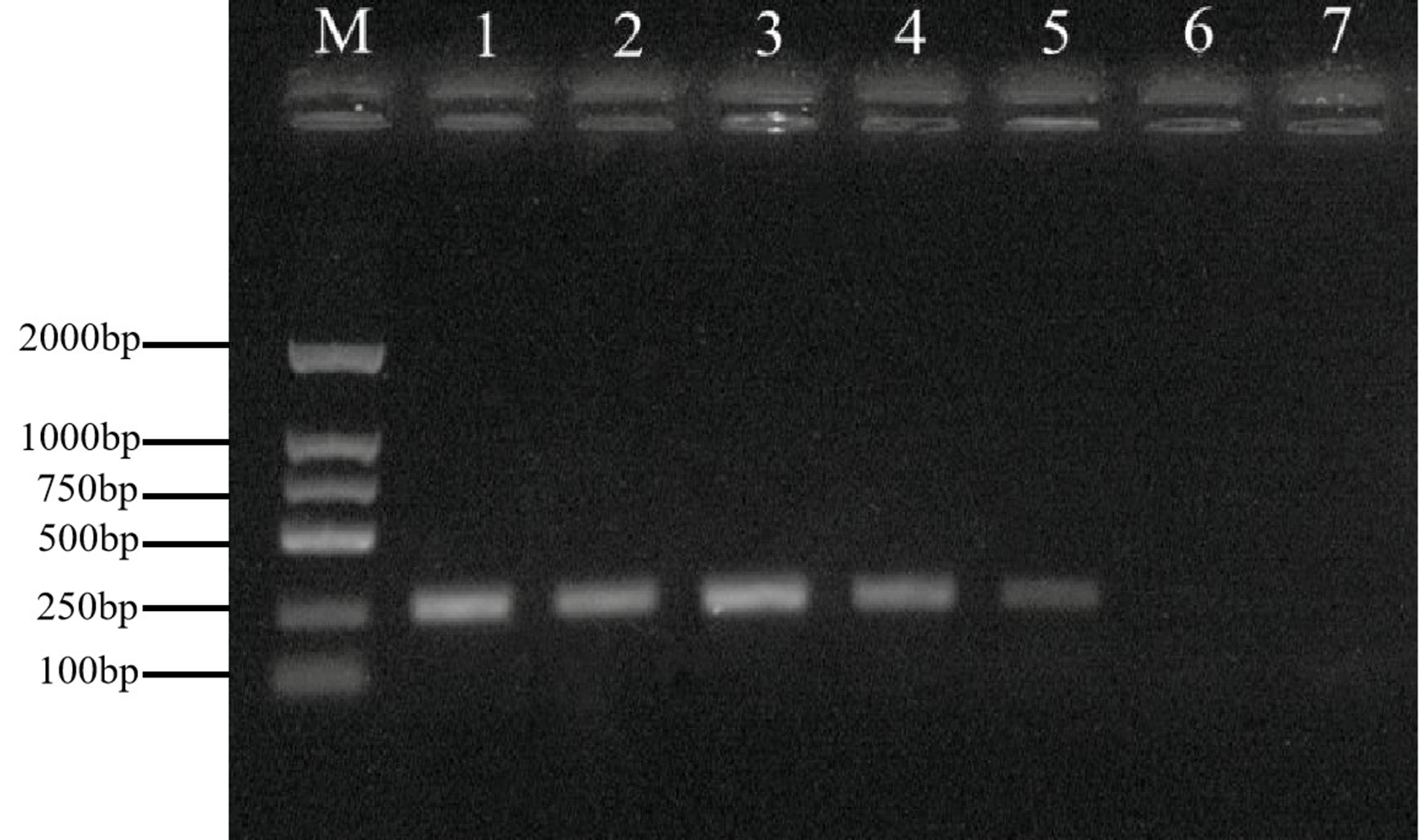

Supplement: Supplementary file 5 [file Image_4.JPEG]
